# Inadequate Mental Health Literacy and Insufficient Physical Activity Potentially Increase the Risks of Anxiety and Depressive Symptoms in Chinese College Students

**DOI:** 10.3389/fpsyt.2021.753695

**Published:** 2021-11-18

**Authors:** Xuexue Huang, Xiaoqing Wang, Jie Hu, Yanni Xue, Yanyan Wei, Yuhui Wan, Xianbing Song, Rui Wang, Bao Zhang, Jun Fang, Shichen Zhang

**Affiliations:** ^1^MOE Key Laboratory of Population Health Across Life Cycle, Department of Maternal, Child and Adolescent Health, School of Public Health, Anhui Medical University, Hefei, China; ^2^Anhui Provincial Key Laboratory of Population Health and Aristogenics, Anhui Medical University, Hefei, China; ^3^Department of Pathology, Anhui Medical College, Hefei, China; ^4^Department of Infectious Disease, First Affiliated Hospital of Anhui Medical University, Hefei, China; ^5^Department of Human Anatomy, Histology and Embryology, Anhui Medical College, Hefei, China; ^6^Information Technology Office, Anqing Medical College, Anqing, China; ^7^Department of Endocrinology, First Affiliated Hospital of Anhui Medical University, Hefei, China; ^8^Department of Clinical Nutriology, First Affiliated Hospital of Anhui Medical University, Hefei, China; ^9^Department of Laboratory of Microbiology, Faculty of Pharmaceutical Science, Sojo University, Kumamoto, Japan; ^10^Department of Preventive Medicine and Public Health Management, Anhui Medical College, Hefei, China

**Keywords:** physical activity, mental health literacy, depressive symptoms, anxiety symptoms, interaction

## Abstract

**Objective:** The present study aimed to examine the interactive associations between physical activity and mental health literacy with anxiety and depressive symptoms in Chinese college students.

**Methods:** A cross-sectional study was conducted from May to July 2020. A total of 7,512 students were recruited from two medical colleges in Hefei and Anqing city in Anhui Province, China. Physical activity, mental health literacy, anxiety and depressive symptoms were measured by self-reported validated instruments. Analyses were conducted with logistic regression models.

**Results:** The prevalence of anxiety and depressive symptoms was 8.6% and 16.4%, respectively. Insufficient physical activity was significantly associated with depressive symptoms (*OR* = 1.359, 95%*CI* 1.184–1.561) and anxiety symptoms (*OR* = 1.492, 95%*CI*: 1.237–1.799). Inadequate mental health literacy was significantly associated with depressive symptoms (*OR* = 3.089, 95%*CI*: 2.607–3.662) and anxiety symptoms (*OR* = 3.675, 95%*CI*: 2.861–4.721). Low physical activity rank (*OR* = 1.438, 95%*CI*: 1.151–1.798) was significantly related with depressive symptoms but not anxiety symptoms. The students who had inadequate mental health literacy and insufficient physical activity had the highest risks of depressive symptoms (*OR* = 5.049, 95% *CI*: 3.649–6.987) and anxiety symptoms (*OR* = 5.270, 95% *CI*: 3.338–8.321).

**Conclusion:** These finding indicated that Chinese college students having insufficient physical activity and inadequate mental health literacy are at risk of exhibiting anxiety and depressive symptoms. Early intervention programs for college students with mental health problems should be considered to enhance their mental health literacy and to increase their physical activities.

## Introduction

The World Health Organization Mental Health Action 2013–2020 put forward that “mental health may be positively influenced by self-confidence and life satisfaction but negatively affected by mental disorders, such as depressive, anxiety and suicide” (1). Depressive and anxiety are the most prevalent group of mental disorders and are responsible for high levels of individual and societal disease burden (2). The prevalence of depressive and anxiety symptoms has increased worldwide. For example, the Healthy Minds Network found that the rates of depressive and anxiety symptoms among college students have raised from 22.0 and 17.2% in 2007, and to 36.6 and 30.9% in 2020 (3). In addition, a large-scale web-based survey for Chinese colleges showed that about 45% of the participants had mental health problems, and the prevalence of depressive and anxiety symptoms were 21.1 and 11.0%, respectively (4). Depressive and anxiety symptoms are common mental health problems experienced by college students that can have an enormous impact on one's interpersonal relationships, quality-of-life, academic difficulties, and working abilities, and in severe cases can lead to suicide, which have been receiving more and more attentions (5, 6). There are a large number of factors underlying depressive and anxiety symptoms. The factors that place college students at risk of depressive and anxiety symptoms are complex and interactive, but the identification of these factors plays an important role in preventing or alleviating depressive and anxiety symptoms.

With the development of activity psychology, physical activity has been widely identified as a protective factor for anxiety and depressive symptoms (7). However, an anonymous web-based survey showed that approximately 52.3% of Chinese college students lack of physical activity, especially high intensity physical activity and specific types of physical activity (e.g., resistance training, stretching) (8). In addition, in 2019 the Youth Risk Behavior Surveillance System (YRBSS) data suggest that, in the United States, less than quarter of students had been physically active as evaluated by ≥60 min/day on all 7 days (23.2%) (9). Research has found that the amount of physical activity has a significant effect on college students' mental health, and different physical activity intensities have significantly different effects on mental health (10). There is also a significant correlation between physical activity and mental health in college students; the higher the degree of physical activity, the higher the mental health (11). Possible reasons may involve that regular physical activity can effectively improve the creativity, happiness, and social ability of inactive students (12). Furthermore, Sheng et al. found that there were interactions among physical activity, self-efficacy, and mental health in middle school students, and that self-efficacy played a mediating role between physical activity and mental health (13). Students with more physical activities have a stronger sense of self-efficacy and tend to have higher subjective well-being and interpersonal adaptability, thereby facilitates the formation of a good mental health state (14).

Mental health literacy (MHL) refers to “knowledge and beliefs about mental disorders which aid their recognition, management or prevention” (15). This definition includes the ability to recognize specific disorders; the knowledge about risk factors and causes of mental health disorders, and how to seek information about a mental health problem; the knowledge to self-treat and to seek of professional help; the attitudes that promote the recognition of mental health problems and seeking appropriate help (15). Inadequate mental health literacy is related to a variety of mental disorders including depressive and anxiety symptoms in college students (16). Previous studies showed that, compared to those with no/mild distress, those with moderate and serious distress had lower recognition of depression and less actions of help-seeking (17). Possible reasons may involve that the inadequacy of an individual's mental health literacy may lead to difficulty in identifying symptoms of mental illness, lack of understanding as well as negative attitude toward professional treatment, thus showing unfavorable attitudes toward seeking professional psychological help as well as poorer adherence to treatment regimens (18). Furthermore, adequate mental health literacy can significantly improve adults' understanding of exercise style and intensity, and promote the recognition of exercise effect, meanwhile, students who perceived the benefits of exercise usually exhibit more physical activities (19, 20).

Although previous studies have reported that physical activity and mental health literacy are independent factors for depression and anxiety (8, 17, 21), the interactive associations of them with anxiety symptoms and depressive symptoms are unclear. The aim of this study was to examine the association between physical activity and mental health literacy with anxiety symptoms and depressive symptoms in Chinese college students. In this regard, we hypothesized that the coexistence of inadequate mental health literacy and lack of physical inactivity would increase the prevalence of anxiety and depressive symptoms in Chinese college students.

## Methods

### Study Design

A cross-sectional study was conducted from May to July 2020, which was approved by the Ethics Committee of Anhui Medical University (approval number 20170290). The sample population was selected by using convenient cluster sampling in Hefei city and Anqing city of Anhui Province, China. Informed consents were obtained from all participants before completing the survey, and all of them could withdraw from the survey at any time without any reason.

An online questionnaire was administered to the students, including socio-demographic variables, physical activity, mental health literacy, depressive symptoms, anxiety symptoms, current cigarette smoking, and alcohol drinking, during 20–30 min session in the classroom. A total of 8,128 participants (mean age of 19 ± 1.11 years) were recruited in this study. Participants were from grade 1 and 2 in the school, resulting in the receipt of 7,512 (92.4%) valid questionnaires (questionnaires with missing data > 5% were discarded).

### Measures

#### General Demographic Information, Cigarette Use and Alcohol Use

The following socio-demographic characteristics were obtained: gender (male or female), grade (freshman or sophomore), registered residence (rural or urban), parents' educational level (< high school degree or ≥high school degree), and self-reported family economy (bad, normal or good). Cigarette use and alcohol use were elicited in two self-report by answering: “During the past 30 days, on how many days did you smoke cigarettes (including traditional smoking and e-cigarettes)?” and “During the past 30 days, on how many days did you have at least one drink of alcohol?” The response options were “yes or no” (21).

#### Physical Activity

Physical activity was assessed with a reliable measure used frequently in the United States as part of the YRBSS (22), and previous study has been demonstrated to have acceptable validity and reliability in the Chinese college students (23, 24). The question is as follows, “On how many of the past 7 days did you do exercises to strengthen or tone your muscles, such as push-ups, sit-ups, or weight lifting?” The responses range from 0 to 7. Subjects with sufficient physical activity were defined as those who exercise at least three days per week. Physical activity rank of the college students was measured by the Physical Activity Rank Scale-3 (PARS-3) (25), and the questions used focused on the physical activity intensity (e.g., “Do you think about the intensity of physical activity?”), activity time (e.g., “How long do you spend each time on physical activity”), and frequency of physical activity (e.g., “How long do your activity every week?”). Physical activity rank was evaluated based on the intensity, time, and frequency of exercise, with the following formula: physical activity rank = frequency × intensity × time. Intensity and frequency are scored 1–5 points, and time is scored 0–4 points, respectively. The present study categorized physical activity rank as low physical activity (≤19 points), medium physical activity (20–42 points), and high physical activity (≥43 points), respectively (25). Previous study suggested a good reliability and validity of for PARS-3 assessing physical activity in Chinese college students (26). The questionnaire showed that the test-retest reliability was high, and the correlation coefficient was *r* = 0.82 (25).

#### Mental Health Literacy

The Adolescent Mental Health Literacy Assessment Questionnaire (AMHLAQ) has been used to measure mental health literacy among college students (27). The questionnaire comprised of 22 items which were grouped as four domains, knowledge (six items), recognition (five items), attitude (six items), and practice (five items). To each question, participants was asked to selected an answer from five categories (strongly disagree, disagree, undecided, agree, strongly agree). The total scores range from 22 to 110 by summing up scores on the 22 items, and higher score indicated a higher level of mental health literacy. A total score <90 points was defined as inadequate mental health literacy. In this study, the Cronbach's α coefficient was 0.911. Verification results for the measurement model were χ^2^/*df* = 19.319, *RMSEA* = 0.069, *AGFI* = 0.881, *NFI* =0.914, *RFI* =0.900, *CFI* = 0.918, and *GFI* = 0.907. This showed that the questionnaire had good validity and reliability (27).

#### Depressive and Anxiety Symptoms

The Center for Epidemiologic Studies Depression Scale (CES-D) was used to evaluate the level of depressive symptoms during the past one week (28). In this study, on a 4-point Likert scale as 0 (rarely or none, < 1 day), 1 (some or a little, 1–2 days), 2 (occasional or moderate, 3–4 days), and 3 (most or all of the time, 5–7 days). A total score more than or equal to 20 points was defined as depressive symptoms. The anxiety symptoms were measured by the Zung Self-Rating Anxiety Scale (SAS), which is a self-reported questionnaire including 20 items. Responses to each question were scored from 1 (not at all or a little time) to 4 (most of the time or all the time) with the total scores ranging from 20 to 80 (29). A total standard score of 50 points was set as a cut-off point of anxiety symptoms. Higher scores on the CES-D and SAS indicated a higher level of depressive and anxiety symptoms. Both the SAS and CES-D have been demonstrated to have acceptable validity and reliability in the Chinese college students (8, 30). In this study, the Cronbach's α coefficient for the CES-D and SAS was 0.859 and 0.763, respectively.

### Statistical Analysis

Statistical analysis was carried out by using SPSS 23.0 (SPSS Inc., Chicago, IL). The Chi-square test was used to compare the prevalence of depressive symptoms and anxiety symptoms among different socio-demographic variables, physical activity, and mental health literacy. Binomial logistic regression models were employed to examine the associations of physical activity, mental health literacy with depressive symptoms, and anxiety symptoms. Multivariate logistic regression models were used to evaluate the interaction of physical activity, mental health literacy with depressive symptoms, and anxiety symptoms. In addition, the data were weighted by gender, and the number of sample population changed when weighted (the weighted subjects were changed from 7,512 to 7,527). Analyses were adjusted for key demographic and socio-demographic variables (e.g., gender, grade, registered residence, parents' educational level, self-reported family economy, cigarette use, and alcohol use). Statistical significance was set at *P* < 0.05.sample population.

## Results

### Characteristics of Participants

Of the 7,527 students, 3,770 were males (50.1%) and 3,757 were females (49.9%). Overall, 1,235 (16.4%), 649 (8.6%) and 5,152 (68.5%) students detected depressive symptoms, anxiety symptoms and insufficient physical activity, respectively. Table 1 showed the prevalence of the depressive symptoms and anxiety symptoms by frequency characteristics. The total rate of depressive symptoms revealed statistically significant differences by grade, mother's educational level, self-reported family economy, cigarette use, and alcohol use (*P* < 0.05 for each). Meanwhile, statistically significant differences were also found in the total rate of anxiety symptoms by gender, father's educational level, self-reported family economy, cigarette use, and alcohol use (*P* < 0.05 for each). In addition, there was a marked difference between depressive symptoms and mental health literacy [inadequate (20.3%) vs. adequate (7.5%), *P* < 0.001), physical activity [insufficient (17.6%) vs. sufficient (13.8%), *P* < 0.001], as well as physical activity rank [low (17.2%) vs. medium (14.8%) vs. high (13.5%)]. Moreover, anxiety symptoms were more likely to occur in college students with inadequate mental health literacy [inadequate (11.0%) vs. adequate (3.2%), *P* < 0.001] and physical inactivity [insufficient (9.5%) vs. sufficient (6.7%), *P* < 0.001]. No significant differences were found for other sociodemographic variables (Table 1).

**Table 1:** Prevalence of depressive symptoms and anxiety symptoms in college students.

**Variable**	**Total Sample** **(***n*** = 7,527)**	**Depressive symptoms** **(***n*** =1,235)**		**Anxiety symptoms** **(***n*** = 649)**	
		***n*** **(%)**	***χ^2^***	***n*** **(%)**	***χ^2^***
Gender			0.114		4.224^*^
Male	3,770 (50.1)	624 (16.6)		350 (9.3)	
Female	3,757 (49.9)	611 (16.3)		299 (8.0)	
Grade			24.080^***^		1.131
Freshman	4,201 (55.8)	767 (18.3)		375 (8.9)	
Sophomore	3,326 (44.2)	467 (14.0)		274 (8.2)	
Registered residence			3.093		1.104
Rural	4,662 (61.9)	762 (16.3)		412 (8.8)	
Urban	2,864 (38.1)	472 (16.5)		237 (8.3)	
Father's educational level			10.127		16.351^**^
<High school degree	5,494 (73.0)	1,032 (17.7)		479 (9.9)	
≥High school degree	2,034 (27.0)	316 (16.3)		170 (9.1)	
Mother's educational level			23.915^***^		8.149
<High school degree	6,196 (82.3)	1,032 (19.0)		542 (9.6)	
≥High school degree	1,330 (17.7)	202 (16.7)		108 (8.8)	
Self-reported family economy			63.690^***^		30.593^***^
Bad	2,886 (38.3)	585 (19.4)		312 (10.8)	
Normal	4,358 (57.9)	617 (14.2)		320 (7.3)	
Good	283 (3.8)	32 (12.6)		17 (6.7)	
Cigarette use			10.194^***^		22.939^***^
No	7,047 (93.6)	1,131 (16.0)		579 (8.2)	
Yes	481 (6.4)	104 (21.6)		70 (14.6)	
Alcohol use			56.758^***^		50.661^***^
No	6,122 (81.3)	910 (14.9)		461 (7.5)	
Yes	1,406 (18.7)	325 (23.1)		189 (13.4)	
Mental health literacy			188.497^***^		124.328^***^
Inadequate	5,233 (69.5)	1,061 (20.3)		577 (11.0)	
Adequate	2,293 (30.5)	173 (7.5)		73 (3.2)	
Physical activity			16.842^***^		16.305^***^
Insufficient	5,152 (68.5)	906 (17.6)		490 (9.5)	
Sufficient	2,374 (31.5)	328 (13.8)		159 (6.7)	
Physical activity rank			9.407^**^		2.316
Low	5,559 (73.9)	954 (17.2)		495 (8.9)	
Medium	1,182 (15.7)	175 (14.8)		90 (7.6)	
High	786 (10.4)	106 (13.5)		64 (8.1)	

*Statistical methods: Chi-square test*;

* *P < 0.05*,

** *P < 0.01*,

*** *P < 0.001; The data were weighted by gender, and the number of sample population changed when weighted*.

### Association of Physical Activity, Mental Health Literacy, Depressive Symptoms With Anxiety Symptoms

As shown in Table 2, depressive symptoms and anxiety symptoms were impacted by mental health literacy and physical activity independently, in Chinese college students. In the models after adjusting for key variables, inadequate mental health literacy was significantly associated with depressive symptoms (*OR* = 3.089, 95% *CI*: 2.607–3.662), and anxiety symptoms (*OR* = 3.675, 95% *CI*: 2.861–4.721). Moreover, while insufficient physical activity was positively correlated with depressive symptoms (*OR* = 1.359, 95% *CI*: 1.184–1.561), and anxiety symptoms (*OR* = 1.492, 95% *CI*: 1.237–1.799), low physical activity rank (*OR* = 1.438, 95% *CI*: 1.151–1.798) was significantly related with depressive symptoms (Table 2). Besides, the interaction of mental health literacy and physical activity had remarkable impact on depressive symptoms and anxiety symptoms (*P* < 0.001 for each). Same associations were also seen in the adjusted models (Table 2).

**Table 2:** Association of physical activity, mental health literacy, depressive symptoms and anxiety symptoms in Chinese college students.

**variable**	**Depressive symptoms**		**Anxiety symptoms**	
	**Crude** ***OR*** **(95% *Cl*)**	**Adjusted** ***OR*** **(95% ***Cl***)^a^**	**Crude *OR* (95% *Cl*)**	**Adjusted** ***OR*** **(95% ***Cl***^a^**
Mental health literacy				
Adequate	1.000	1.000	1.000	1.000
Inadequate	3.113 (2.629–3.685)^***^	3.089 (2.607–3.662)^***^	3.786 (2.951–4.858)^***^	3.675 (2.861–4.721)^***^
Physical activity
Sufficient	1.000	1.000	1.000	1.000
Insufficient	1.331 (1.161–1.526)^***^	1.359 (1.184–1.561)^***^	1.459 (1.212–1.757)^***^	1.492 (1.237–1.799)^***^
Physical activity rank				
High	1.000	1.000	1.000	1.000
Medium	1.118 (0.862–1.450)	1.145 (0.879–1.491)	0.929 (0.665–1.296)	0.986 (0.703–1.383)
Low	1.331 (1.072–1.653)^**^	1.438 (1.151–1.798)^**^	1.099 (0.838-1.441)	1.243 (0.939-1.644)
Mental health literacy × Physical activity				
Adequate × Sufficient	1.000	1.000	1.000	1.000
Adequate × Insufficient	2.291 (1.894-2.771)^***^	2.333 (1.7925–2.827)^***^	2.982 (2.237–3.976)^***^	2.934 (2.197–3.919)^***^
Mental health literacy × Physical activity rank				
Adequate × High	1.000	1.000	1.000	1.000
Adequate × Medium	2.814 (1.948–4.065)^***^	2.790 (1.926–4.042)^***^	2.436 (1.517–3.913)^***^	2.324 (1.443–3.741)^***^
Adequate × Low	2.831 (2.340–3.426)^***^	2.938 (2.421–3.565)^***^	3.682 (2.754–4.925)^***^	3.650 (2.722–4.894)^***^

*OR is odds ratio; Cl is confidence interval*.

** *P < 0.01*,

*** *P < 0.001*.

a *Adjusted for gender, grade, registered residence, parents' educational level, self-reported family economy, cigarette use, alcohol use*.

### The Interactions of Physical Activity, Mental Health Literacy, Depressive Symptoms With Anxiety Symptoms

**Figure 1:**
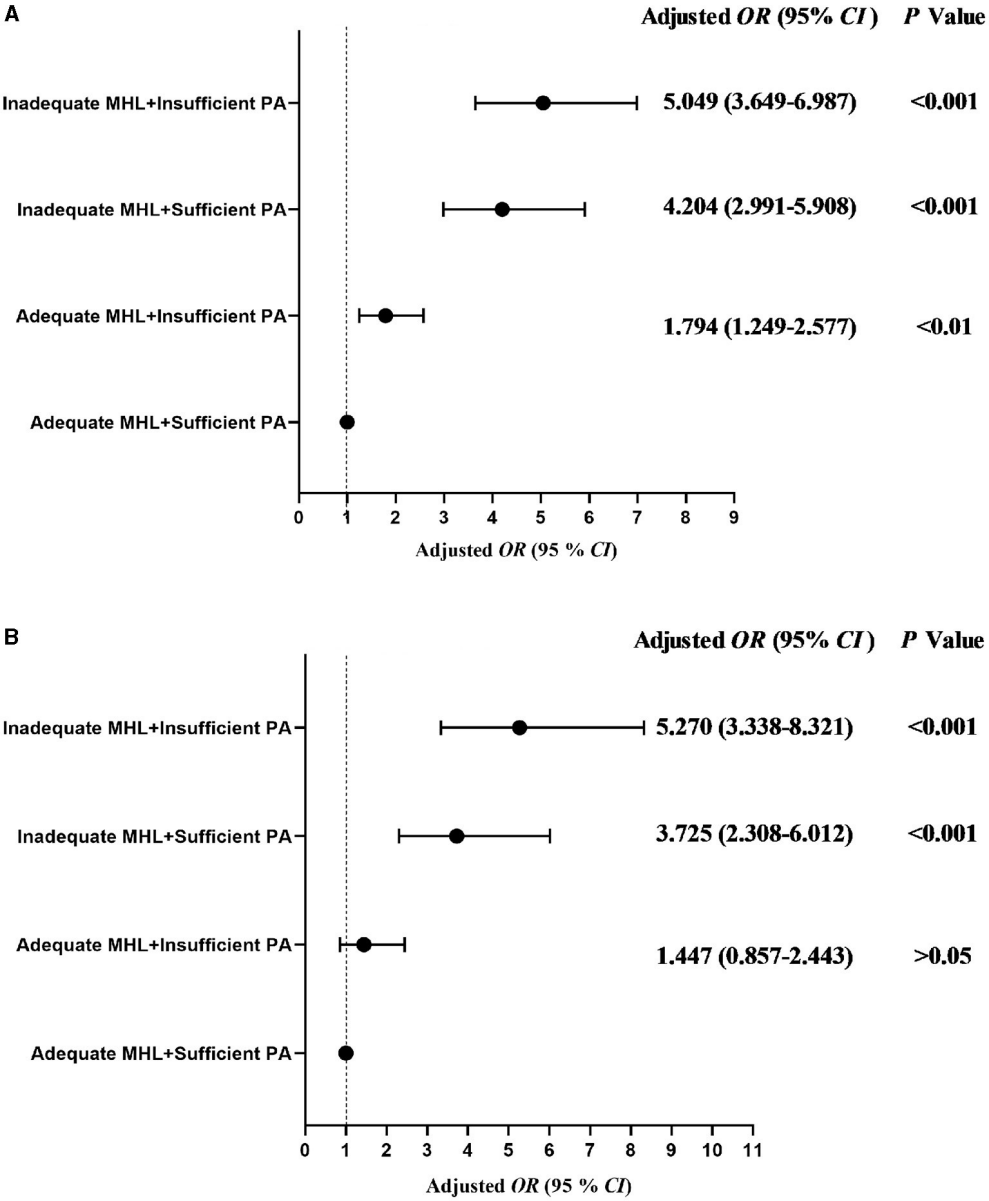
Odds ratio (95% *CI*) associated with the interactions of physical activity, mental health literacy, depressive symptoms and anxiety symptoms in college students. **(A)** Depressive symptoms, **(B)** Anxiety symptoms. MHL, mental health literacy; PA, physical activity; *OR*, odds ratio; *CI*, confidence interval. Adjusted for gender, grade, registered residence, parents' educational level, self-reported family economy, cigarette use, alcohol use.

**Figure 2:**
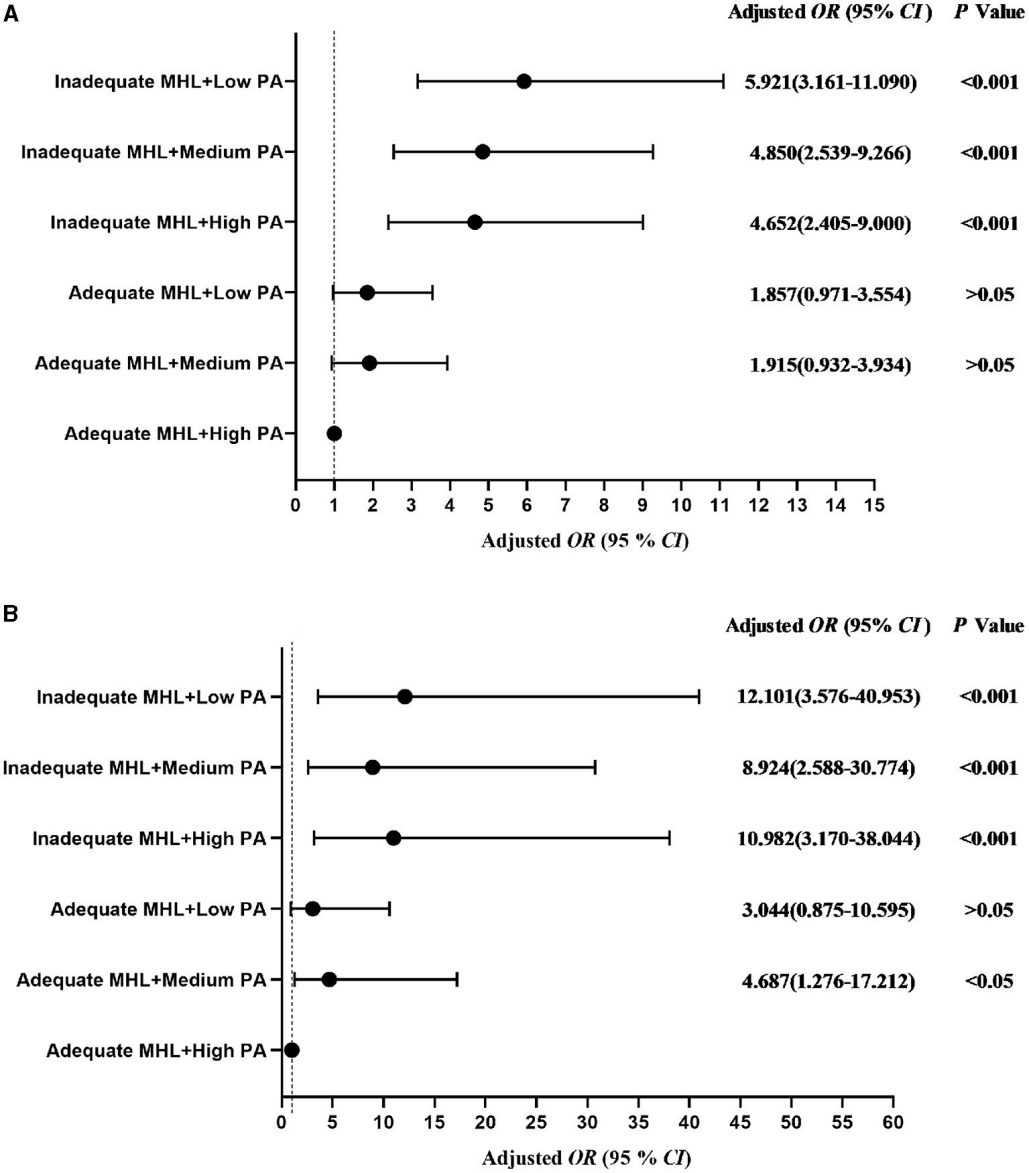
Odds ratio (95% *CI*) associated with the interactions of physical activity rank, mental health literacy, depressive symptoms and anxiety symptoms in college students. **(A)** Depressive symptoms, **(B)** Anxiety symptoms. MHL, mental health literacy; PA, physical activity; *OR*, odds ratio; *CI*, confidence interval. Adjusted for gender, grade, registered residence, parents' educational level, self-reported family economy, cigarette use, alcohol use.

Figure 1 showed the relationship of the different groups of mental health literacy and physical activity with anxiety symptoms and depressive symptoms in all students. The adjusted *OR* (95% *CI*) was described for each group in comparison with the reference group (adequate mental health literacy and sufficient physical activity) for anxiety symptoms and depressive symptoms, respectively. The students with inadequate mental health literacy and insufficient physical activity had the highest risk of anxiety symptoms (*OR* = 5.270, 95% *CI*: 3.338–8.321, *P* < 0.001) and depressive symptoms (*OR* = 5.049, 95% *CI*: 3.649–6.987, *P* < 0.001; Figure 1). Same associations were also seen in the adjusted models (Figure 2). For more details check Table A1 in Appendix A.

## Discussion

In the current study, we detected the prevalence of anxiety symptom was 8.6%, and the depressive symptom was 16.4% in the selected college students, which was lower than the results with nation-wide survey of college students (11.0, and 21.1%, respectively) (4). The inconsistent results may be related to the different evaluation criterion as well as the different origin of the participants. Moreover, our survey was conducted in late May and early July, the COVID-19 epidemic situation in the investigation area has been effectively controlled to a certain extent, and most college students have adapted to living at home, which may also be a reason of the lower rate of the depressive and anxiety symptoms (31). Furthermore, we found that freshmen had significantly higher prevalence of depressive symptoms than sophomore, the possible explanation could be a selection of especially vulnerable personalities (e.g., high performers), lack of adaptability to new environment, vulnerability due to the transitional period from high school to college and strain due to the highly competitive environment caused by the high entry requirements (32). Our results indicated anxiety symptoms and depressive symptoms were associated with low family economic status, cigarette and alcohol use, which was similarly reported in previous studies (33, 34).

The proportion of college students with insufficient physical activity in this study was 68.5%, which is higher than data from previous study (64.7%) (35), which may be related to the fact that the investigation time is during the epidemic period of COVID-19 confinements. A systematic review reported significant reduction of varying degree in walking, moderate, vigorous, and total physical activity levels during the COVID-19 pandemic lockdown in college students (36). In recent years, the positive benefits of physical activity for mental health have been widely recognized (33, 37). In response to the urgent need to improve physical activity levels, many countries including China have adopted positively physical activity strategies. Consequently, the level of physical activity has been increasing (38, 39). Although our study found an insignificant relationship between low physical activity rank and anxiety symptoms, sufficient physical activity was found to be significantly correlated with anxiety and depressive symptoms. A previous study in undergraduate students has reported that students who engaged in medium and high levels of physical activity had lower depressive symptoms than those with a low level of physical activity (8), which is in agreement with our research.

Several hypotheses have been proposed to explain the inverse association between physical activity and mental health problems (40). Regular physical activity helps to restore or preserve proper function of the nervous system by stimulating the cerebral cortex, and simultaneously increasing the supply of oxygen and nutrients, all of which contribute to improve cognitive and mental health (41). Alternative or additional mechanisms involve that physical activity may enhance psychosocial determinants of mental health including self-efficacy and sense of mastery through effective interaction with the social context (42). The experience of physical activity can facilitate the interaction with the natural environment, and potentially promote emotion which provides an opportunity to confirm their own capabilities (42). Correspondingly, compelling evidence showed increased benefits of moderate-to-vigorous physical activity on psychological health (8, 43). Thus, encouraging college student to participate in physical activity may contribute to improve mental health and reduce risk of mental disorders.

Meanwhile, our study also provides important information with regard to mental health literacy in Chinese college students. Mental health literacy is critical for early recognition and intervention in mental disorders (19). A growing number of studies have shown that inadequate mental health literacy is a strong risk factor of depression and anxiety (17, 44). Our results indicated that those reported adequate mental health literacy had a reduced risk of anxiety and depressive symptoms than those reported inadequate mental health literacy. Interestingly, our results confirmed that students with adequate mental health literacy as well as sufficient physical activity had the lowest risk of depressive symptoms and anxiety symptoms, and highest risk was found in those with both inadequate mental health literacy and insufficient physical activity. Namely, our results suggested a synergistic effect of inadequate mental health literacy and insufficient physical activity in increasing the risk of mental problems in college students. Individuals with a better-developed health literacy have skills and competences that enable them to engage in various personal health promoting behaviors (e.g., regular physical activity) (45). As the extension of the concept of health literacy, mental health literacy has similar functions, which could increase the level of physical activity in students through improving the in-depth understanding of perceived exercise benefit (15, 20). Addition, past researches that have revealed self-efficacy may not always directly affect physical activity, instead acting in conjunction with behavioral capability (knowledge and/or skill) variables, such as health literacy (26, 46). Furthermore, higher health literacy levels strengthened the positive relationship between self-efficacy and health behaviors (47). Consequently, we speculate that mental health literacy can also facilitate the interaction with the self-efficacy, and significantly promote weekly physical activity. The findings from our study appear to bear this out. Wang et al. found a clear, the demands for psychological knowledge and interventions during COVID-19 epidemic in students with anxiety and depression symptom was significantly higher than those without (48). This may be due to college students with higher mental health literacy may better understand the contributing factors or the triggers of psychological problems, thus having less psychological distress (49). Furthermore, mental health literacy is highly correlated with positive attitudes toward help-seeking (50), and attitudes toward help-seeking is associated with mental health (51). This finding suggests a potential mechanism regarding the link between mental health literacy and mental health.

## Strengths and Limitations

There are some limitations in this study. Firstly, we used a cross-sectional study design, so causal relationships were not defined. Longitudinal or prospective cohort design are needed to further clarify the causality among these variables. Secondly, we used self-reported data, therefore, recall and reporting bias cannot be excluded. Thirdly, the subjects recruited from two medical colleges, and the proportion of male and female students was not coordinated, so it should be careful to generalize our findings to all Chinese college students. Despite the above limitations, the present study first highlights the importance of associations of mental health literacy and physical activity with depressive symptom and anxiety symptom in Chinese college students. In addition, the data were collected during the COVID-19 pandemic, it is thus an important reference s for the study of Chinese college students' mental health problems during the epidemic period of COVID-19.

## Conclusion

Taken together, our results indicated that mental health literacy and physical activity and their interactions were related to depressive symptoms and anxiety symptom among Chinese college students. Thus, physical activity and mental health literacy should be considered in developing intervention programs for the aim to reduce the rate of mental health problems among college students.

## Data Availability Statement

The original contributions presented in the study are included in the article/Supplementary Material, further inquiries can be directed to the corresponding authors.

## Ethics Statement

The studies involving human participants were reviewed and approved by Ethics Committee of Anhui Medical University (approval number 20170290). Written informed consent to participate in this study was provided by the participants' legal guardian/next of kin. Written informed consent was obtained from the individual(s), and minor(s)' legal guardian/next of kin, for the publication of any potentially identifiable images or data included in this article.

## Author Contributions

SZ and YHW were responsible for the conception and design of the study. XH, JH, YX, YYW, XS, RW, and BZ were involved in data collection. XW and XH conducted the statistical analysis. All authors contributed to interpretation of the findings. XH and XW wrote the first draft of the paper, which was critically revised by SZ and JF. SZ, XW, and JF provided funding for the project. The final manuscript was approved by all authors.

## Funding

This work was supported by the National Natural Science Foundation of China (81402699 and 81573512), the Natural Science Foundation in Higher Education of Anhui (KJ2020A0209) and the Domestic Visiting Study Project for Outstanding Young Talents in Colleges and Universities in Anhui Province in 2021 (gxgnfx2021183). The funder had no role in study design, data collection and analysis, decision to publish, or preparation of the manuscript.

## Conflict of Interest

The authors declare that the research was conducted in the absence of any commercial or financial relationships that could be construed as a potential conflict of interest.

## Publisher's Note

All claims expressed in this article are solely those of the authors and do not necessarily represent those of their affiliated organizations, or those of the publisher, the editors and the reviewers. Any product that may be evaluated in this article, or claim that may be made by its manufacturer, is not guaranteed or endorsed by the publisher.
