# Supplementary material for: Inadequate Mental Health Literacy and Insufficient Physical Activity Potentially Increase the Risks of Anxiety and Depressive Symptoms in Chinese College Students
Source: Front Psychiatry. 2021 Nov 18;12:753695. doi: 10.3389/fpsyt.2021.753695 (PMC8637166; doi:10.3389/fpsyt.2021.753695)
Supplement: Supplementary file 1 [file Table_1.pdf]

APPENDIX A

TABLE A1 Odds ratio (95 % *CI*) associated with the interactions of physical activity, mental health literacy, depressive symptoms and anxiety symptoms in college students.

Variable		Depressive symptoms			Anxiety symptoms		
		Crude *OR* (95%*CI*)	Adjusted *OR* (95%*CI*)[a]	$P^{\#}$	Crude *OR* (95%*CI*)	Adjusted *OR* (95%*CI*)[a]	$P^{\#}$
Mental health literacy	Physical activity			<0.001			<0.001
Adequate	Sufficient	1.000	1.000		1.000	1.000	
	Insufficient	1.808 (1.261-2.593)**	1.794 (1.249-2.577)**		1.449 (0.860-2.443)	1.447 (0.857-2.443)	
Inadequate	Sufficient	4.158 (2.965-5.831)***	4.204 (2.991-5.908)***		3.853 (2.392-6.208)***	3.725 (2.308-6.012)***	
	Insufficient	4.967 (3.596-6.860)***	5.049 (3.649-6.987)***		5.338 (3.386-8.415)***	5.270 (3.338-8.321)***	
Mental health literacy	Physical activity rank			<0.001			<0.001
Adequate	High	1.000	1.000		1.000	1.000	
	Medium	1.887 (0.923-3.857)	1.915 (0.932-3.934)		4.371 (1.194-15.994)*	4.687 (1.276-17.212)*	
	Low	1.876 (0.987-3.564)	1.857 (0.971-3.554)		2.891 (0.835-10.012)	3.044 (0.875-10.595)	
Inadequate	High	4.619 (2.400-8.892)***	4.652 (2.405-9.000)***		11.455 (3.317-39.558)***	10.982 (3.170-38.044)***	
	Medium	4.894 (2.574-9.306)***	4.850 (2.539-9.266)***		9.078 (2.641-31.206)***	8.924 (2.588-30.774)***	
	Low	5.819 (3.127-10.832)***	5.921 (3.161-11.090)***		11.521 (3.419-38.817)***	12.101 (3.576-40.953)***	

Note. OR is odds ratio; CI is confidence interval; $^{*}P<0.05$, $^{**}P<0.01$, $^{***}P<0.001$ *compared with reference.*

[a] *Adjusted for gender, grade, registered residence, parents' educational level, self-reported family economy, cigarette use, alcohol use.*

[#] *P-value of interaction between physical activity and mental health literacy on depressive symptoms and anxiety symptoms in multiplicative model.*
